# Probability and Statistics with Applications in Finance and Economics

**DOI:** 10.1155/2015/618785

**Published:** 2015-08-25

**Authors:** Sarah Brown, Wing Keung Wong

**Affiliations:** ^1^Department of Economics, University of Sheffield, 9 Mappin Street, Sheffield S1 4DT, UK; ^2^Department of Economics, Hong Kong Baptist University, WLB, Shaw Campus, Kowloon Tong, Hong Kong

Probability and statistics play a vital role in every field of human activity. In particular, they are quantitative tools widely used in the areas of economics and finance. Knowledge of modern probability and statistics is essential for the development of economic and finance theories and for the testing of their validity through robust analysis of real-world data. For example, probability and statistics could help to shape effective monetary and fiscal policies and to develop pricing models for financial assets such as equities, bonds, currencies, and derivative securities. The importance of developing robust methods for such empirical analysis has become particularly important following the recent global financial crisis in 2008, which has placed economic and finance theories under the spotlight.

In this connection, this special issue has been co-edited by the following guest editors Sarah Brown (University of Sheffield), Terence Chong (The Chinese University of Hong Kong), Pulak Ghosh (Indian Institute of Management), Tai Zhong Hu (University of Science and Technology of China), Lu Lin (Shandong University), Wing-Keung Wong (Hong Kong Baptist University), and Zhijie Xiao (Boston College) brings together high quality papers that are relevant for academics and practitioners alike from a range of disciplines including economics, finance, and statistics. This special issue is devoted to advancements in the applications of probability and statistics in the areas of economics and finance bringing together practical, state-of-the-art applications of probability, and statistical techniques in economics and finance. We hope that the papers published in this special edition will stimulate further research in this area.

The papers published in the special issue collectively make important contributions to a wide range of areas including contributions which enhance our understanding of financial and commodity markets as well as contributions to statistical theory, which may open up important avenues for applied analysis in the future. To be specific, with respect to finance applications, G. Hinterleitner et al. (2015) explore market structure at the opening of the trading day and its influence on subsequent trading. Their experimental framework compares a single continuous double auction and two complement markets with different call auction designs as opening mechanisms. Their findings indicate that a call auction not only improves market efficiency and liquidity at the beginning of the trading day when compared to the stand-alone continuous double auction, but also causes positive spillover effects on subsequent trading. On the other hand, G. M. Goerg (2014) presents a parametric, bijective transformation to generate heavy tail versions of arbitrary random variables. The Lambert W function is used to model and remove heavy tails from continuous random variables using a data-transformation approach. Motivated by the observation that financial data is frequently characterised by negative skewness and excess kurtosis, the simulations and applications to S&P 500 log-returns in this paper importantly demonstrate the usefulness of the introduced methodology. Given that natural gas has become an extremely important commodity for the global economy, a commodity which is characterised by an increasingly risky and volatile market, J. Tang et al. (2015) focus on estimating the risk of natural gas portfolios using a GARCH-EVT-copula model. The findings indicate the importance of conducting further research on dependence structure in the market for this critical commodity.

With respect to purely theoretical contributions, C.-W. Lin et al. (2014) demonstrate that, under suitable conditions, an almost sure central limit theorem for self-normalized products of sums of partial sums holds under a fairly general growth condition on the weight sequence, an important statistical result. Finally, C. Yin et al. (2014) focus on one of the key topics of actuarial science and finance, namely, the optimal dividend problem. Specifically, they explore the optimal dividends problem for a company whose cash reserves follow a general Lévy process with certain positive jumps and arbitrary negative jumps. In this interesting contribution, they present conditions under which the optimal dividend strategy, among all admissible ones, takes the form of a barrier strategy.

*Sarah Brown* *Sarah Brown* *Wing Keung Wong* *Wing Keung Wong*

